# *Purpureocillium lilacinum* for plant growth promotion and biocontrol against root-knot nematodes infecting eggplant

**DOI:** 10.1371/journal.pone.0283550

**Published:** 2023-03-24

**Authors:** Masudulla Khan, Kiwamu Tanaka

**Affiliations:** 1 Department of Botany, Section of Plant Pathology/Nematology, Aligarh Muslim University, Aligarh, UP, India; 2 Botany Section, Women’s College, Aligarh Muslim University, Aligarh, UP, India; 3 Department of Plant Pathology, Washington State University, Pullman, WA, United States of America; Indian Agricultural Research Institute, INDIA

## Abstract

*Purpureocillium lilacinum* is a biocontrol Ascomycota fungus against various plant pathogens. In the present study, the efficacy of *P*. *lilacinum* was evaluated against a root-knot nematode, *Meloidogyne incognita* that infects eggplants. We performed an in vitro experiment in which the direct effects of *P*. *lilacinum* on the second-stage juvenile survival and egg hatching of *M*. *incognita* were tested at different exposure times. The results showed that *P*. *lilacinum* significantly reduced the rates of egg hatching and juvenile survival in a dose-dependent manner. Microscopic observation demonstrated that *P*. *lilacinum* directly penetrated the eggs and contacted the juveniles, indicating how *P*. *lilacinum* parasitizes *M*. *incognita*. We also performed a pot assay in which soil-grown eggplants were treated with *P*. *lilacinum* followed by inoculation with *M*. *incognita*. The results indicated that *P*. *lilacinum* effectively reduced the nematode population and the number of galls in plant roots. Interestingly, application of *P*. *lilacinum* resulted in significant enhancements in plant growth and biomass, even under nematode infection, while it improved plant photosynthetic pigments, i.e., chlorophyll and carotenoids. Taken together, our study suggested that *P*. *lilacinum* can be used as a plant growth-promoting fungus and a biological nematicide for disease management of root-knot nematodes in eggplants.

## Introduction

Eggplant, *Solanum melongena* L., also known as brinjal in Indian subcontinents or aubergine in Europe, belongs to the family Solanaceae and is one of the most important vegetable crops. Eggplants are susceptible to many diseases including soilborne diseases causing great losses in yield and quality. In general, diseases caused by soilborne pathogens are difficult to control, and plant resistance to many soilborne pathogens has not been fully studied [1–3]. Plant-parasitic nematodes are also not an exception to soilborne pathogens, causing high losses in the agriculture sector for eggplants [4].

Root-knot nematodes, *Meloidogyne* spp., are soil-borne sedentary endoparasites and are one of the most important plant-parasitic nematodes. Root-knot nematodes decrease crop yield by up to 75% in the heavily infested fields [5, 6]. The nematodes invade the plant roots with the help of stylet and migrate intercellularly and develop the feeding site where they initiate the formation of giant cells known as galls or knots [7]. Generally, chemical pesticides and soil fumigation are used for plant disease management on a large scale in the agriculture sector. Heavy use of toxic chemicals causes human health issues and environmental toxicity. To reduce the use of synthetic chemicals, it is urgently needed to develop alternative control methods that have minimal impact on the environment. Over recent years, biocontrol methods and biotechnological approaches have been widely studied and applied for disease management caused by plant-parasitic nematodes [8, 9].

*Purpureocillium lilacinum* (formerly *Paecilomyces lilacinus*) is a nematophagous fungus that belongs to the Ascomycota phylum [10, 11]. This fungus occurs naturally in the soil and rhizospheres of many crops [12, 13]. It can grow at a wide range of temperatures (8–38°C) and pH tolerances [14, 15]. *Purpureocillium lilacinum* forms a dense mycelium from which conidiophores arise. Spores germinate when suitable conditions, e.g., moisture and nutrients are met [14, 16]. This soil fungus has been extensively tested for the biocontrol of plant-parasitic nematodes [17, 18]. It has also been reported as an effective biocontrol of different species of insects, e.g., cotton aphids, western flower thrips, glasshouse red spider mites, greenhouse whiteflies, and leaf-cutting ants [19–21]. Recently, the fungus was evaluated as a biocontrol agent against some fungal plant pathogens, such as *Sclerotinia sclerotiorum*, *Verticillium dahliae*, *Phytophthora capsici*, and *P*. *infestans* [22–24]. These reports suggest that *P*. *lilacinum* is one of the most promising biocontrol agents against plant pathogens and pests.

In the present study, the efficacy of *P*. *lilacinum* was evaluated for plant protection against *M*. *incognita*, which causes root-knot disease in eggplants. Based on in vitro experiments and greenhouse trials, our data demonstrated that *P*. *lilacinum* significantly alleviates infection of the root-knot nematode and enhances eggplant growth, suggesting that *P*. *lilacinum* is a useful biocontrol agent for disease management of root-knot nematodes in eggplants.

## Materials and methods

### Fungal culture and inoculum preparation

**Fig 1:**
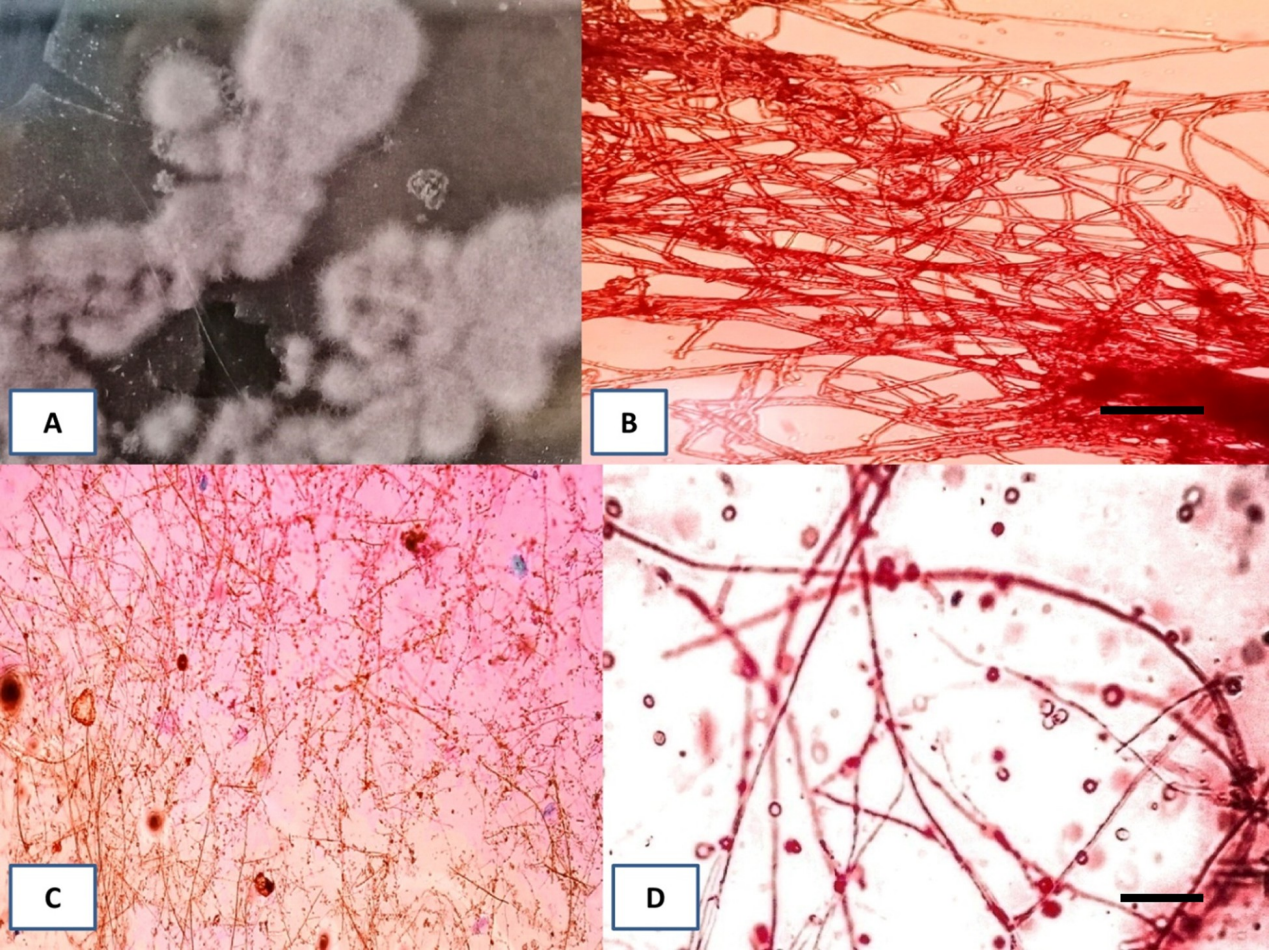
Representative pictures of *P*. *lilacinum*. (A) Fungus culture on PDA media. (B) Mycelium of fungi. Scale bar = 25 μm (C and D) Mycelium and conidia of fungi. Scale bars = 50 μm.

*Purpureocillium lilacinum* was obtained from the Indian Type Culture Collection at the Indian Agriculture Research Institute (New Delhi, India). Culturing of *P*. *lilacinum* was performed as described in our previous study [25]. Subculturing of fungus was performed on potato dextrose agar (PDA) medium for use in the experiment. Richard’s liquid medium was used to obtain the inoculum of the fungus [26], which contained 10 g/L of potassium nitrate, 5 g/L of potassium dihydrogen phosphate, 2.5 g/L of magnesium sulfate, 0.02 g/L of ferric chloride, and 50 g/L of sucrose. Each 80 mL of the Richard’s liquid medium was placed in Erlenmeyer flasks followed by sterilization at 103.4 kPa for 15 min in an autoclave machine. The flasks were incubated at 25 ± 1°C for ~15 days on a incubator shaker with 100 rpm (Fig 1). For inoculum preparation, the fungal mycelia mat on filter paper was washed in sterile water, and extra water and nutrients were removed with blotting paper. Ten grams of mycelia mat (fungal inoculum) was mixed in 100 mL of distilled water followed by blending in a Waring blender (10,000 RPM) for 30 s. The inoculum collected was adjusted to 10^8^ CFU/mL and labeled as a standard suspension (S), and consecutive concentrations, such as S/2, S/10, S/25, and S/50, were prepared using distilled water. Ten milliliters of standard (S) suspension were used to inoculate eggplants in a pot assay.

### Propagation of *Meloidogyne incognita*

**Fig 2:**
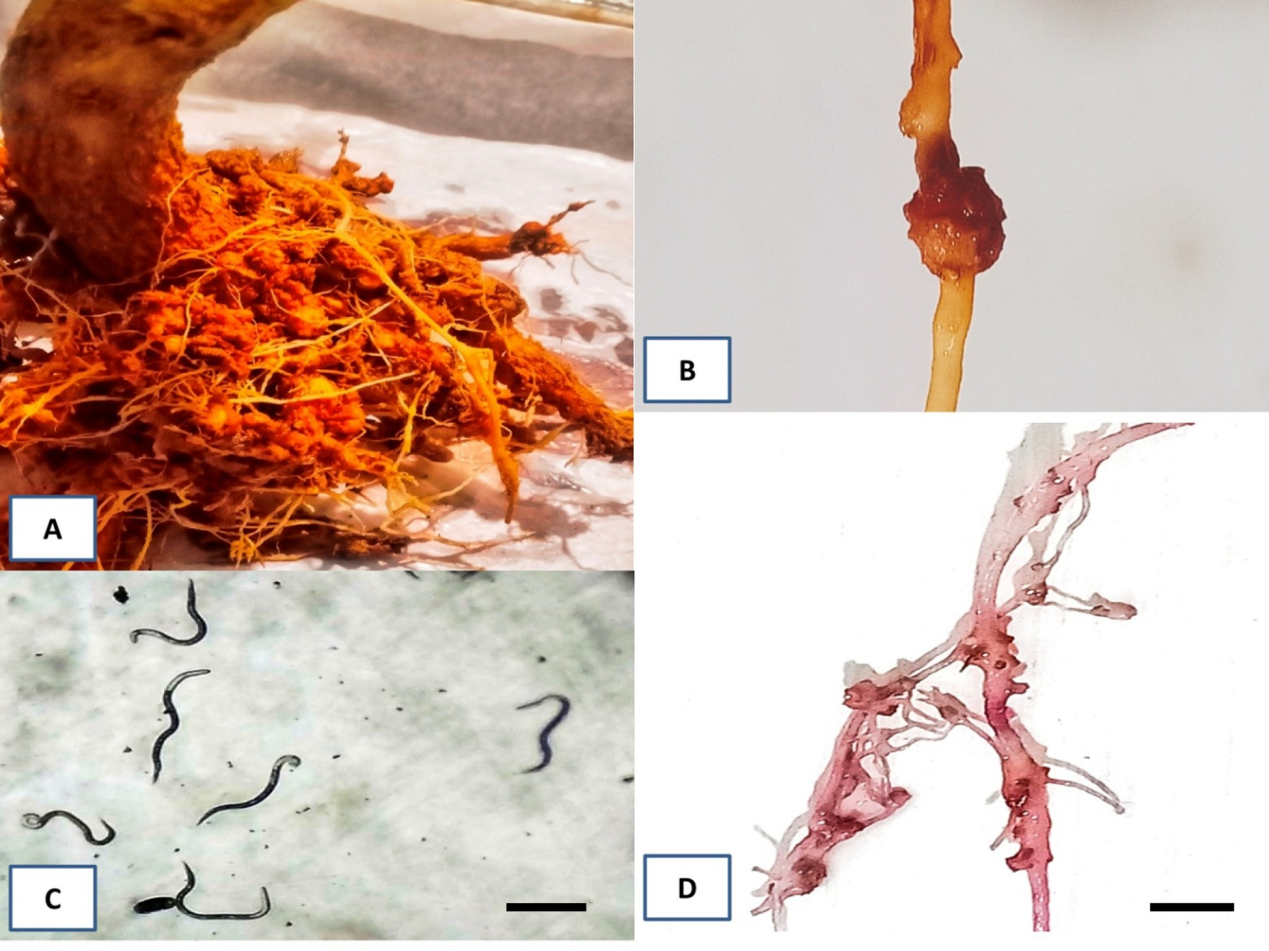
Representative pictures of disease symptoms caused by juveniles of the root-knot nematode *M*. *incognita*. (A) Large root galls or knots were formed throughout the root system of eggplants infected by *M*. *incognita*. (B) A closed up picture of a root gall. (C) Juveniles in J2 stage. Scale bar = 200 μm. (D) Egg mass on infected roots. Scale bar = 10 mm.

The root-knot nematode *M*. *incognita* was isolated from infected eggplant roots and maintained in greenhouse for research purpose [27]. Egg masses were hand-picked with the help of sterilized forceps from infected roots. The isolated egg masses were washed with distilled water and placed in a small sieve of 9-cm diameter with 1-mm pore size containing layers of tissue paper. The sieve was placed in a Petri plate containing distilled water deep enough to contact the egg masses and these assemblies were kept in an incubator at 25 ± 1°C for the hatching of second-stage juveniles (J2). Hatched J2 were collected and the volume of the nematode suspension was adjusted to 200 ± 5 nematodes per mL. Twenty mL of the nematode suspension (4000 freshly hatched J2) was added to each pot [27]. The plants were harvested 90 days post-inoculation, and the roots were cut into 4–5 cm pieces after washing to collect eggs. The eggs were hatched in water, and active J2 were collected (Fig 2).

### Mortality bioassay

To measure the efficacy of *P*. *lilacinum* on the mortality of *M*. *incognita*, 10 mL of fungal suspension with different concentrations (S, S/5, S/10, S/20, S/50, S/100) was placed in each empty Petri dish. Then, 100 freshly hatched J2s were added to each Petri dish. The dishes were incubated at 25 ± 1°C in a biochemical oxygen demand (BOD) incubator, and the effect on mortality was observed after 48 h intervals.

### Egg hatching assay

To perform the egg-hatching assay, 10 mL of fungal culture suspension of different concentrations (S, S/5, S/10, S/20, S/50, S/100) was placed in each Petri dish. Three eggs masses of the same size were placed in each Petri dish. The rates of nematode hatching were observed after incubation at 27–28°C for 48 h by counting the number of hatched juveniles under the microscope. Each treatment was replicated five times at room temperature.

### Microscopic observation of the effect of the fungi on nematode eggs and juveniles

*Purpureocillium lilacinum* was incubated in the center of a Petri dish containing PDA medium amended with streptomycin at 10 mg/L and incubated at 25 ± 2°C for 10 days. After incubation, 100 surface-sterilized *M*. *incognita* eggs were placed on the dish (the eggs were sterilized with 2% sodium hypochlorite solution for 5 minutes beforehand). After 10 days, the eggs were stained with cotton blue, and the percentage of egg parasitism was evaluated by counting the parasitized and non-parasitized eggs under a microscope. The eggs with either direct hyphal penetration or disintegration of their contents were considered dead [28], while eggs that contained live juveniles and eggs hatching juveniles were counted as viable.

### Inoculation of *P*. *lilacinum* with *M*. *incognita*-infected eggplants in a greenhouse

A greenhouse experiment was conducted to evaluate the antagonistic properties of *P*. *lilacinum* against the root-knot nematode *M*. *incognita* infecting eggplants. The eggplant seeds (cv. VNR-218) were obtained from VNR Nursery Pvt. Ltd. (Chhattisgarh, India). Before sowing, eggplant seeds were surface sterilized with 0.1% sodium hypochlorite solution for 1 min and then washed with distilled water. Two-week-old eggplant seedlings were planted in plastic pots (30-cm diameter) containing 3.5 kg of sterilized sandy loam soil collected from the field of A.M.U. (Aligarh, India). The collected soil was autoclaved at 137.9 kPa for 20 min. Inoculum of *P*. *lilacinum* (10^8^ CFU/mL) was applied at 10 mL/pot as a soil drench. Second-stage juveniles of *M*. *incognita* (4000 J2s/pot) were added one day after the fungal inoculation. Three treatments of pots were prepared with five replicates for each treatment, including a control, as follows: C, control = no fungi and nematodes; M, inoculated with *M*. *incognita*; and M+P, inoculated with *M*. *incognita* and *P*. *lilacinum*.

### Measurement of plant growth and disease evaluation

Plants were collected 90 days after nematode inoculation. The plant growth criteria, including plant height, plant weight, and shoot dry weight, were measured. Plants were uprooted carefully. Plant height was measured in cm from the end of the root up to the top of the first leaf. To determine the plant’s fresh weight, excess water was removed by blotting before weighing. Plants were kept in envelopes at 60°C for 2–3 days before dry weight determination. The roots were washed gently with tap water to separate the soil particles and were stained with acid fuchsin in lactic acid to evaluate the phases of the nematode developmental stages, females, galls, and egg masses under binuclear conditions [29]. Juveniles of nematodes were extracted from the soil using a modified Baermann technique and counted using a stereoscopic microscope with a counting slide [30]. Two hundred fifty grams of subsampled well-mixed soil from each treatment was processed by Cobb’s sieving and decanting technique followed by Baermann funnel extraction to obtain nematodes [31]. Every 24 h, the nematode suspension was collected, and the numbers of nematodes were counted in five aliquots of 1 mL of suspension from each sample. The population of nematodes per kg of soil was calculated from the means of five counts. For the estimation of photosynthetic metabolites in plant leaves, the chlorophyll and carotenoid contents were measured based on a previously published method [32].

### Data analysis

Sigma Plot was used for the preparation of graphs of the mean value with the standard error. All experiments were repeated at least five times, and the data obtained were analyzed using ANOVA followed by the Tukey–Kramer multiple comparison test. A difference with P < 0.05 was considered significant.

## Results

### Direct effects of *P*. *lilacinum* on egg hatching and juvenile viability of *M*. *incognita* under in vitro conditions

**Fig 3:**
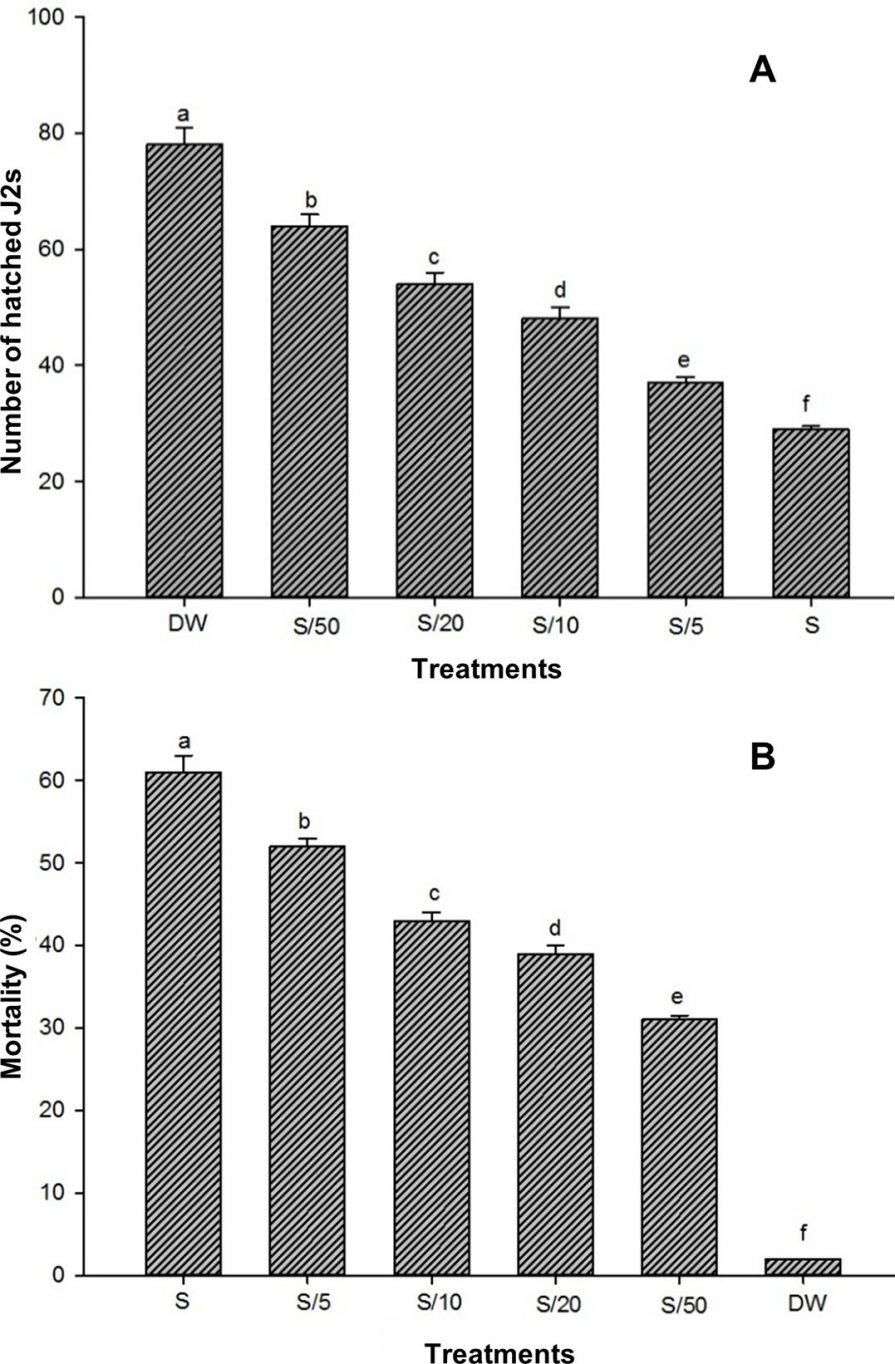
Effects of *P*. *lilacinum* on egg hatching and juvenile viability of *M*. *incognita*. The eggs or J2s of *M*. *incognita* were incubated with suspension cultures of *P*. *lilacinum* in petri dishes at different concentrations with serial dilution (S, S/5, S/10, S/20, S/50, S/100), where S is 10 g mycelia in 100 mL distilled water. (A) The number of hatched juveniles was measured to count the rates of egg hatching after 48 h of incubation. (B) The mortality of J2 *M*. *incognita* was measured after 48 h of incubation. Different letters indicate statistically significant differences at P < 0.05 (n = 5). DW, distilled water control.

Direct exposure of *M*. *incognita* to *P*. *lilacinum* was performed to test whether there were any inhibitory effects on egg hatching and juvenile viability of *M*. *incognita*. To this end, the eggs and J2s of *M*. *incognita* were incubated with suspension cultures of *P*. *lilacinum* at different concentrations with serial dilution (S, S/5, S/10, S/20, S/50, S/100), where S is 10 g mycelia in 100 mL distilled water. As shown in Fig 3, *P*. *lilacinum* showed high inhibitory activities against *M*. *incognita*, while the effects became more prominent upon increasing the exposure time and concentration. Egg hatching was reduced as the concentration of *P*. *lilacinum* increased. Maximum hatching occurred after 48 hours, and the highest inhibition of egg hatching was observed at the (S) standard concentration with 62.8% less than control in hatching (Fig 3A). The concentration of S/5 caused 52.5%, S/10 caused 38.4%, S/20 caused 30.7% and S/50 caused a 17.9% reduction in hatching after 48 h (Fig 3A). Regarding the effect of *P*. *lilacinum* on nematode viability, the highest mortality (61%) was observed in the standard (S) suspension of *P*. *lilacinum* (Fig 3B), indicating that juvenile mortality was greatly influenced by the concentration of fungal inoculum. The lowest mortality (31%) was observed at the S/50 concentration, which was the lowest fungal concentration we tested (Fig 3B). These results demonstrated that *P*. *lilacinum* directly inhibited the egg hatching and the juvenile viability of *M*. *incognita*.

### *P*. *lilacinum* parasitizes eggs and juveniles of *M*. *incognita*

**Fig 4:**
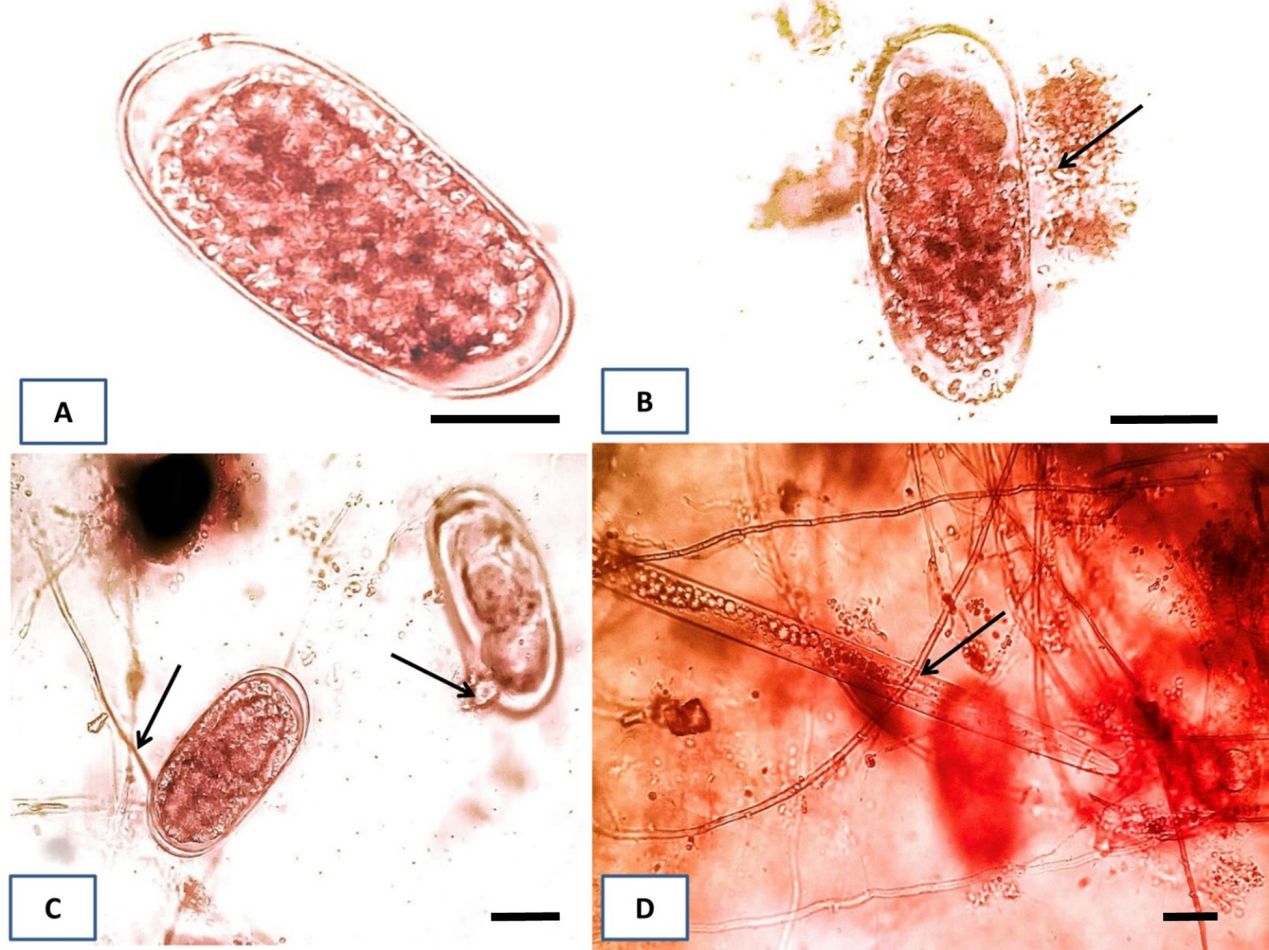
Direct effects of *P*. *lilacinum* on *M*. *incognita* eggs and juveniles. Microscopic observation was performed during the incubation of *P*. *lilacinum* with *M*. *incognita* eggs (B and C) and juveniles (D) on PDA agar plates. A intact egg was shown in (A). Arrows indicate penetration and aggregation of fungal conidia and mycelia in eggs (B and C) and attaching to a J2 juvenile (D). Scale bars = 50 μm.

We then observed the penetration and infection of fungi (Fig 4). Egg parasitism was recorded after ten days of fungal exposure to eggs, and the presence of mycelium and spores inside or outside the eggs was considered to indicate infection. The fungi were also observed penetrating hatching eggs (Fig 4B and 4C). After ten days, almost all eggs were found infected by fungi. Additionally, the fungal hyphae directly interacted with and reduced the juveniles’ mortality (Fig 4D). These results demonstrated how *P*. *lilacinum* parasitizes the eggs and juveniles of *M*. *incognita*.

### Effect of *P*. *lilacinum* on plant growth of pot-grown eggplants

**Fig 5:**
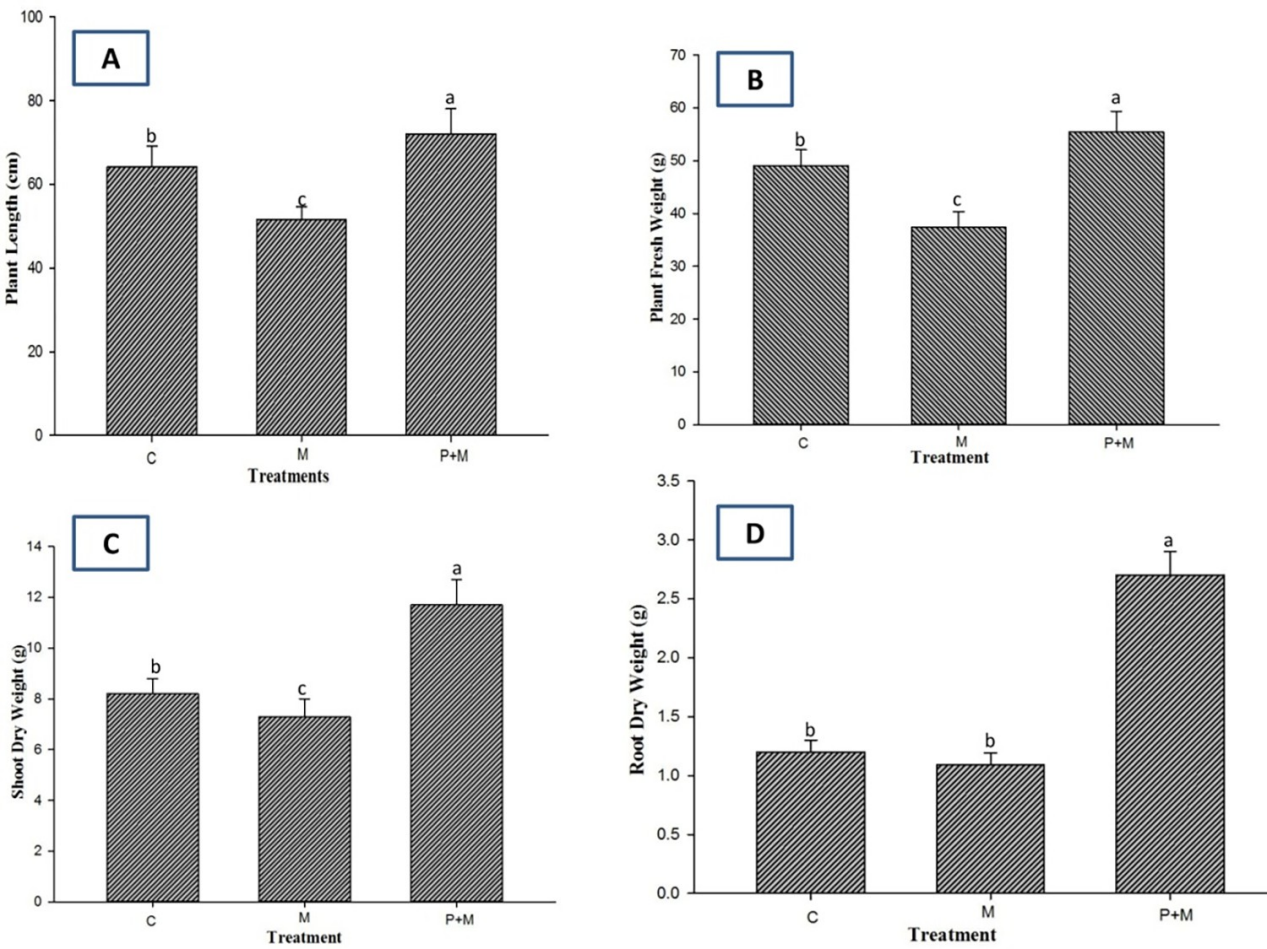
Effect of *P*. *lilacinum* on plant growth of eggplants infected by *M*. *incognita*. The plants were inoculated with*M*. *incognita* with (P+M) or without (M) preinoculation with *P*. *lilacinum*. No inoculation of either *M*. *incognita* or*P*. *lilacinum* was used as a control (C). Plants were collected 90 days after nematode inoculation to measure plant height (A), fresh weight (B), shoot dry weight (C), and root dry weight (D). Different letters indicate statistically significant differences at P < 0.05 (n = 5).

**Fig 6:**
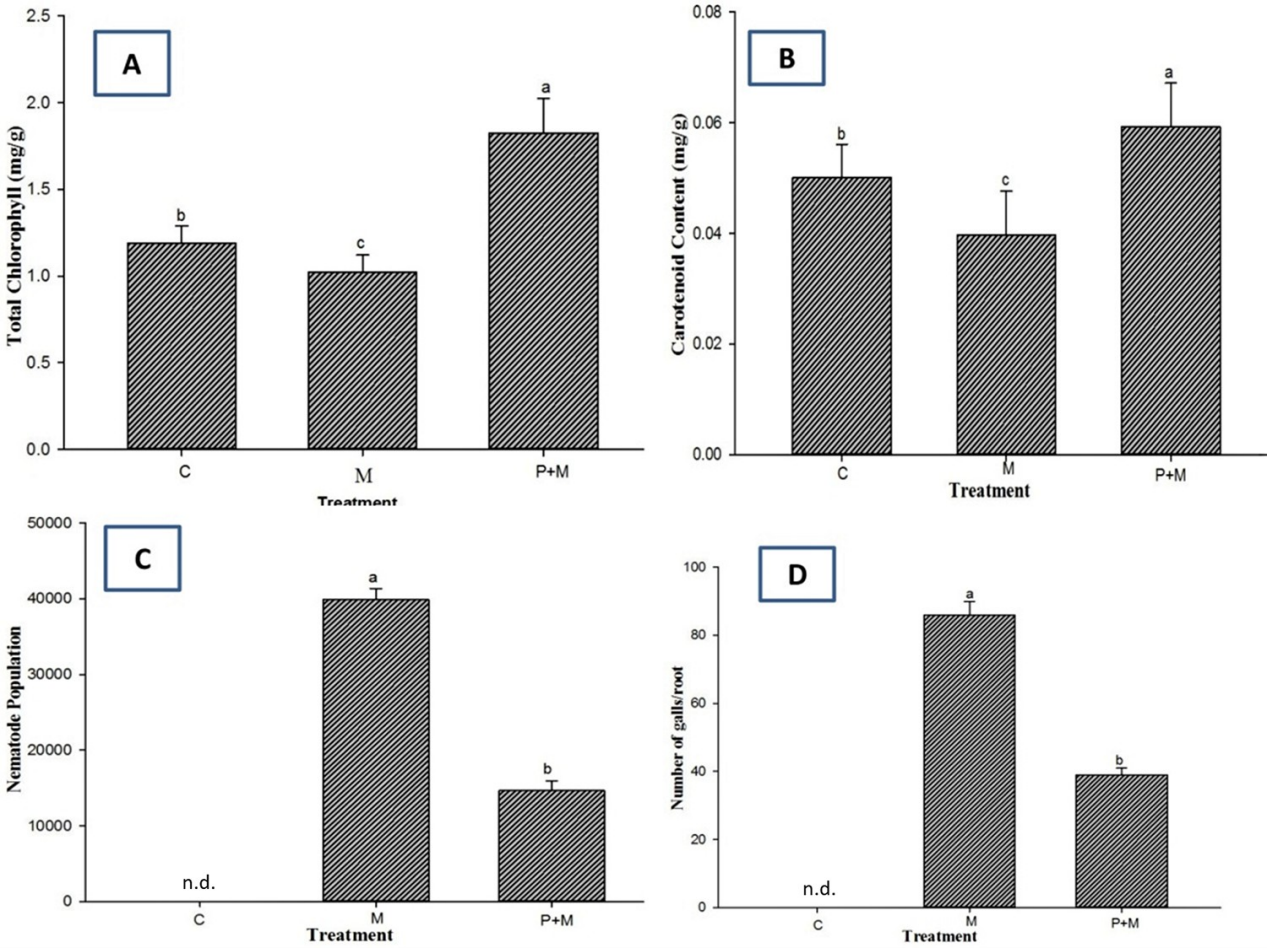
Effects of *P*. *lilacinum* on photosynthetic pigments and nematode propagation in eggplants infected by *M*. *incognita*. The plants were inoculated with *M*. *Incognita* with (P+M) or without (M) preinoculation with *P*. *lilacinum*. No inoculation of either M. incognita or*P*. *Lilacinum* was used as a control as labeled “C”. Plants were collected 90 days after nematode inoculation to measure total chlorophyll (A) and carotenoids (B) as well as the nematode population (C) and the numbers of galls per root (D). Different letters indicate statistically significant differences at P < 0.05 (n = 5) and “n.d.” represents “not detected”.

**Fig 7:**
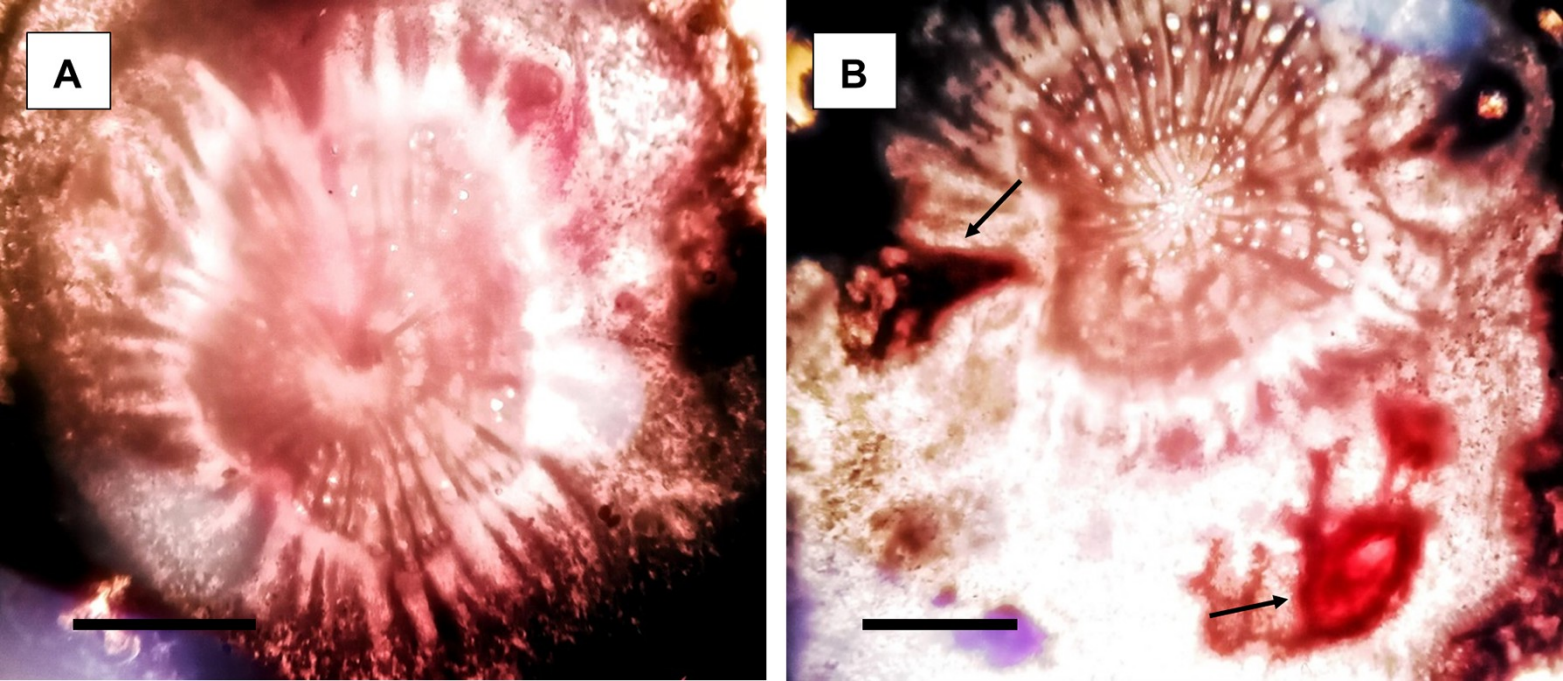
*P*. *lilacinum* mitigates the damage to root tissues caused by *M*. *incognita*. Transverse section of the eggplant roots inoculated with *M*. *incognita* with (A) or without (B) preinoculation of *P*. *lilacinum*. The arrows indicate the females of *M*. *incognita*. present in the root tissue. Bar = 0.5 mm.

The inoculation of *M*. *incognita* (“M” in Fig 5) inhibited the growth of the soil-grown eggplants; for example, there was a 19.7% reduction in plant height and a 23.8% reduction in plant fresh weight. Significant reductions in plant biomass, for example, a 12.2% reduction in shoot dry weight and a 9.1% reduction in root dry weight, were also found after the inoculation of *M*. *incognita* compared to the uninoculated plants (“C” in Fig 5). In contrast, the application of *P*. *lilacinum* (“P+M” in Fig 5) mitigated the adverse effects caused by *M*. *incognita* infection and further enhanced plant growth in terms of height and biomass (Fig 5A–5D). In the same manner, *P*. *lilacinum* significantly improved the chlorophyll and carotenoid contents in plants (Fig 6A and 6B). This result suggests that *P*. *lilacinum* can be used as a plant growth-promoting fungus as proposed previously [8]. Finally, we found that the application of *P*. *lilacinum* caused significant reductions of 62% and 52% in the gall numbers and the nematode population, respectively (Fig 6C and 6D). Transverse sections of the eggplant roots showed that the presence of female *M*. *incognita* was less frequently observed when *P*. *lilacinum* was preinoculated onto the eggplant roots (Fig 7). Taken together, our results suggested that *P*. *lilacinum*, which directly acts on *M*. *incognita*, can be useful as an effective nematicide to protect eggplants against root-knot nematodes.

## Discussion

The present study investigated the biocontrol fungus *P*. *lilacinum* for plant protection against plant-parasitic nematodes. This fungus is one of the biocontrol agents approved for use against nematodes in farms and commercial fields. However, information regarding its efficacy is scarce in different plants, e.g., eggplants.

*Purpureocillium lilacinum* employs flexible lifestyles, such as soil saprobes, plant endophytes, and nematode pathogens. The fungus was proposed as a nematode egg-pathogenic fungus, but several papers have demonstrated that it infects all life stages of root-knot nematodes [33, 34]. The timing of fungal application has also been studied. For example, Dahlin et al. recommended a sequential application of a chemical pesticide (e.g., fluopyram) followed by *P*. *lilacinum* application since it was highly effective to control the nematodes and increasing the crop yields in comparison to a single application of one or other of two [35]. Currently, the fungus is commercially used as a biological nematicide (e.g., MeloCon, by Certis USA LLC) against a wide range of nematodes, including root-knot, cyst, burrowing, reniform, root lesion, and false root nematodes [36]. *Purpureocillium lilacinum* is getting popular since it is more cost-effective and environmentally sound when compared to synthetic nematicides [37].

In the present study, we observed based on the in vitro experiment that *P*. *lilacinum* directly penetrated hatched eggs and juveniles of *M*. *incognita*, which are infectious for eggplants. The data showed that *P*. *lilacinum* reduced at most 62.8% and 61% in the egg hatching rate and the juvenile mortality rate of *M*. *incognita*, respectively. Our results were comparable with the previous study using two different strains of *P*. *lilacinum* (local strain PLA and PLB and commercial strain PLM) that exhibited 66.0–78.8% parasitism on eggs and 88–89% reduction in the egg hatching [38]. Our microscopic observation demonstrated that *P*. *lilacinum* directly penetrated the eggs and contacted the juveniles, indicating how the fungus parasitizes *M*. *incognita*. These results demonstrated how the fungus breaks into the nematode body to parasitize or directly kill and how the fungus reduced the rates of egg hatching and juvenile survival.

We demonstrated that a preinoculated soil with *P*. *lilacinum* reduced *M*. *incognita* root galling by 52% and egg masses by 62% on eggplants. These data in eggplants were comparable with that seen in tomato for which Kiewnick et al. [39] reported 45% reduction in the galling index and 69% reduction in the number of egg masses per root system after preinoculation with *P*. *lilacinum* strain 251 (another commercial strain). Taken together, *P*. *lilacinum* is the effective biocontrol agent against *M*. *incognita* infecting various plants, including eggplants.

What could be the possible molecular mechanisms by which *P*. *lilacinum* parasitizes *M*. *incognita*? The genomic sequence of *P*. *lilacinum* revealed enriched contents of carbohydrate-active enzymes (CAZymes), proteases, secondary metabolites, and pathogenesis-related genes in comparison to other fungi [40]. In particular, it has been demonstrated that cuticle-degrading protease, chitinase, and serine protease, play important roles in degrading nematode eggshells [41–43] and thereby *P*. *lilacinum* parasitizes *M*. *incognita*. In addition, another studies reported that the proteins with CFEM (common in fungal extracellular membrane) domains were up-regulated during the infection of nematode eggs and suggested that the CFEM proteins is important for the recognition of nematode-eggs by the fungus [44, 45]. Recently, *P*. *lilacinum* was shown to produce secondary metabolites (e.g., leucinostatins, paecilomide, and acremoxanthones) that cause strong mortality and inhibit nematode reproduction. These metabolites could be another mechanism that kills nematodes [46–48].

Our data demonstrated that *P*. *lilacinum* promotes plant growth in eggplants. This positive effect could be because *P*. *lilacinum* alleviated the adverse effect of the nematode infection on the plant growth. However, *P*. *lilacinum* has been shown to have beneficial effects on maize, common bean, and soybean plants, where nitrogen and phosphate availabilities were increased in soil [13]. Interestingly, our data showed that *P*. *lilacinum* promoted the accumulation of photosynthetic pigments, i.e., chlorophyll and carotenoids, which could be attributed to increased nutrient availability in the soil, as reported previously [13].

In conclusion, *P*. *lilacinum* is an effective biocontrol agent against the root-knot nematode *M*. *incognita* infecting eggplants, while it provides plant growth promotion. The fungus has been well studied in terms of its molecular and ecological characteristics. In the future, in-depth studies of the direct beneficial effects of the fungus on plant physiology and immune responses will be needed. This type of research is important because a lack of knowledge of how this biocontrol agent works on plants may deprive growers of a potentially effective application strategy. In addition, some strains of *P*. *lilacinum* could pose a medical hazard affecting on human and animal health due to its potential mycoses of the lungs, heart, eyes, and skin, e.g., hyalohyphomycosis [49]. Although some *P*. *lilacinum* strains can be pathogenic to animals, including humans, the risk of infection through the current mode of application is likely to be very low. Nevertheless, it is always important to conduct thorough risk assessments to ensure the safety of human health and the environment, as is the case with other biocontrol agents and biopesticides [50].

## References

[1] Whipps JM. Developments in the Biological Control of Soil-borne Plant Pathogens. In: Callow JA, editor. Advances in Botanical Research. Academic Press; 1997. pp. 1–134. doi: 10.1016/S0065-2296(08)60119-6

[2] Mora-Romero GA, Félix-Gastélum R, Bomberger RA, Romero-Urías C, Tanaka K. Common potato disease symptoms: ambiguity of symptom-based identification of causal pathogens and value of on-site molecular diagnostics. J Gen Plant Pathol. 2022;88: 89–104. doi: 10.1007/s10327-021-01045-2

[3] Braley LE, Jewell JB, Figueroa J, Humann J, Main D, Mora-Romero GA, et al. Nanopore sequencing with GraphMap for comprehensive pathogen detection in potato field soil. Plant Disease. 2023 [cited 28 Feb 2023]. doi: 10.1094/PDIS-01-23-0052-SR 36724099

[4] Fatima S, Khan F, Asif M, Alotaibi SS, Islam K, Shariq M, et al. Root-Knot Disease Suppression in Eggplant Based on Three Growth Ages of *Ganoderma lucidum*. Microorganisms. 2022;10: 1068. doi: 10.3390/microorganisms10051068 35630510 PMC9144836

[5] Liang J, Zheng J. Advances of studies on biological control of vegetable root-knot nematode in installation cultivation. Chin Agric Sci Bull. 2010;26: 290–293.

[6] Mesa-Valle CM, Garrido-Cardenas JA, Cebrian-Carmona J, Talavera M, Manzano-Agugliaro F. Global Research on Plant Nematodes. Agronomy. 2020;10: 1148. doi: 10.3390/agronomy10081148

[7] Siddique S, Grundler FM. Parasitic nematodes manipulate plant development to establish feeding sites. Current Opinion in Microbiology. 2018;46: 102–108. doi: 10.1016/j.mib.2018.09.004 30326406

[8] Poveda J, Abril-Urias P, Escobar C. Biological Control of Plant-Parasitic Nematodes by Filamentous Fungi Inducers of Resistance: *Trichoderma*, Mycorrhizal and Endophytic Fungi. Frontiers in Microbiology. 2020;11. Available: https://www.frontiersin.org/articles/10.3389/fmicb.2020.00992 doi: 10.3389/fmicb.2020.00992 32523567 PMC7261880

[9] Bali S, Zhang L, Franco J, Gleason C. Biotechnological advances with applicability in potatoes for resistance against root-knot nematodes. Current Opinion in Biotechnology. 2021;70: 226–233. doi: 10.1016/j.copbio.2021.06.010 34217954

[10] Prasad P, Varshney D, Adholeya A. Whole genome annotation and comparative genomic analyses of bio-control fungus *Purpureocillium lilacinum*. BMC Genomics. 2015;16: 1004. doi: 10.1186/s12864-015-2229-2 26607873 PMC4658809

[11] Girardi NS, Sosa AL, Etcheverry MG, Passone MA. In vitro characterization bioassays of the nematophagous fungus *Purpureocillium lilacinum*: Evaluation on growth, extracellular enzymes, mycotoxins and survival in the surrounding agroecosystem of tomato. Fungal Biology. 2022;126: 300–307. doi: 10.1016/j.funbio.2022.02.001 35314061

[12] Barbosa BB, Pimentel JP, Rodovalho NS, Bertini SCB, Kumar A, Ferreira LFR, et al. Ascomycetous isolates promote soil biological and nutritional attributes in corn and soybeans in sandy and clayey soils. Rhizosphere. 2022;24: 100625. doi: 10.1016/j.rhisph.2022.100625

[13] Baron NC, Pollo A de S, Rigobelo EC. *Purpureocillium lilacinum* and *Metarhizium marquandii* as plant growth-promoting fungi. PeerJ. 2020;8: e9005. doi: 10.7717/peerj.9005 32518715 PMC7261125

[14] Samson RA. Paecilomyces and some allied hyphomycetes. Studies in Mycology. 1974;6: 1–119.

[15] Domsch KH, Anderson T-H, Gams W. Compendium of soil fungi. Reprint der Ausg. von 1980. Berlin: IHW-Verlag; 1993.

[16] Thom C. Cultural studies of species of *Penicillium*. Washington, D.C.: Bulletin of the U.S. Department of Agriculture, Bureau Animal Industry; 1910.

[17] Atkins SD, Clark IM, Pande S, Hirsch PR, Kerry BR. The use of real-time PCR and species-specific primers for the identification and monitoring of *Paecilomyces lilacinus*. FEMS Microbiology Ecology. 2005;51: 257–264. doi: 10.1016/j.femsec.2004.09.002 16329874

[18] Hajji L, Hlaoua W, Regaieg H, Horrigue-Raouani N. Biocontrol Potential of Verticillium leptobactrum and *Purpureocillium lilacinum* Against *Meloidogyne javanica* and *Globodera pallida* on Potato (*Solanum tuberosum*). Am J Potato Res. 2017;94: 178–183. doi: 10.1007/s12230-016-9554-0

[19] Fiedler Ż, Sosnowska D. Nematophagous fungus *Paecilomyces lilacinus* (Thom) Samson is also a biological agent for control of greenhouse insects and mite pests. BioControl. 2007;52: 547–558. doi: 10.1007/s10526-006-9052-2

[20] Goffré D, Folgarait PJ. *Purpureocillium lilacinum*, potential agent for biological control of the leaf-cutting ant *Acromyrmex lundii*. J Invertebr Pathol. 2015;130: 107–115. doi: 10.1016/j.jip.2015.07.008 26205173

[21] Elbanhawy AA, Elsherbiny EA, Abd El-Mageed AE, Abdel-Fattah GM. Potential of fungal metabolites as a biocontrol agent against cotton aphid, *Aphis gossypii* Glover and the possible mechanisms of action. Pesticide Biochemistry and Physiology. 2019;159: 34–40. doi: 10.1016/j.pestbp.2019.05.013 31400782

[22] Yang F, Abdelnabby H, Xiao Y. A mutant of the nematophagous fungus *Paecilomyces lilacinus* (Thom) is a novel biocontrol agent for *Sclerotinia sclerotiorum*. Microbial Pathogenesis. 2015;89: 169–176. doi: 10.1016/j.micpath.2015.10.012 26521137

[23] Lan X, Zhang J, Zong Z, Ma Q, Wang Y. Evaluation of the Biocontrol Potential of *Purpureocillium lilacinum* QLP12 against *Verticillium dahliae* in Eggplant. BioMed Research International. 2017;2017: e4101357. doi: 10.1155/2017/4101357 28303252 PMC5337802

[24] Hu J, Hou S, Li M, Wang J, Wu F, Lin X. The better suppression of pepper *Phytophthora* blight by arbuscular mycorrhizal (AM) fungus than *Purpureocillium lilacinum* alone or combined with AM fungus. J Soils Sediments. 2020;20: 792–800. doi: 10.1007/s11368-019-02438-9

[25] Khan M, Khan AU, Rafatullah M, Alam M, Bogdanchikova N, Garibo D. Search for Effective Approaches to Fight Microorganisms Causing High Losses in Agriculture: Application of *P*. *lilacinum* Metabolites and Mycosynthesised Silver Nanoparticles. Biomolecules. 2022;12: 174. doi: 10.3390/biom12020174 35204674 PMC8961611

[26] Riker AJ, Riker RS. Introduction to research on plant diseases. Swift, St. Louis. 1936.

[27] Khan M, Siddiqui ZA. Interactions of *Meloidogyne incognita*, *Ralstonia solanacearum* and *Phomopsis vexans* on eggplant in sand mix and fly ash mix soils. Scientia Horticulturae. 2017;225: 177–184. doi: 10.1016/j.scienta.2017.06.016

[28] Khan A, Williams KL, Nevalainen HKM. Control of plant-parasitic nematodes by *Paecilomyces lilacinus* and *Monacrosporium lysipagum* in pot trials. Biocontrol. 2006;51: 643–658. doi: 10.1007/s10526-005-4241-y

[29] Byrd DW, Kirkpatrick JRT, Barker KR. An Improved Technique for Clearing and Staining Plant Tissues for Detection of Nematodes. Journal of Nematology. 1983;14: 142–143. PMC2618249 19295781

[30] Hooper DJ, Hallmann J, Subbotin SA. Methods for extraction, processing and detection of plant and soil nematodes. Plant parasitic nematodes in subtropical and tropical agriculture. 2005; 53–86. doi: 10.1079/9780851997278.0053

[31] Southey JF, editor. Laboratory methods for work with plant and soil nematodes. 6th ed. London: HMSO; 1986.

[32] Mackinney G. Absorption of light by chlorophyll solutions. Journal of biological chemistry. 1941;140: 315–322.

[33] Yang F, Abdelnabby H, Xiao Y. The role of a phospholipase (PLD) in virulence of *Purpureocillium lilacinum* (*Paecilomyces lilacinum*). Microbial Pathogenesis. 2015;85: 11–20. doi: 10.1016/j.micpath.2015.05.008 26026833

[34] Sarven MS, Aminuzzaman FM, Huq MdE. Dose-response relations between *Purpureocillium lilacinum* PLSAU-1 and *Meloidogyne incognita* infecting brinjal plant on plant growth and nematode management: a greenhouse study. Egyptian Journal of Biological Pest Control. 2019;29: 26. doi: 10.1186/s41938-019-0128-6

[35] Dahlin P, Eder R, Consoli E, Krauss J, Kiewnick S. Integrated control of Meloidogyne incognita in tomatoes using fluopyram and *Purpureocillium lilacinum* strain 251. Crop Protection. 2019;124: 104874. doi: 10.1016/j.cropro.2019.104874

[36] Arthurs S, Dara SK. Microbial biopesticides for invertebrate pests and their markets in the United States. Journal of Invertebrate Pathology. 2019;165: 13–21. doi: 10.1016/j.jip.2018.01.008 29402394

[37] Abd-Elgawad MMM. Optimizing biological control agents for controlling nematodes of tomato in Egypt. Egypt J Biol Pest Control. 2020;30: 58. doi: 10.1186/s41938-020-00252-x

[38] Pau CG, Leong CTS, Wongt SK, Eng L, Kundat R. Isolation of Indigenous Strains of Paecilomyces lilacinus with. Int J Agric Biol. 2012;14.

[39] Kiewnick S, Neumann S, Sikora RA, Frey JE. Effect of *Meloidogyne incognita* Inoculum Density and Application Rate of *Paecilomyces lilacinus* Strain 251 on Biocontrol Efficacy and Colonization of Egg Masses Analyzed by Real-Time Quantitative PCR. Phytopathology®. 2011;101: 105–112. doi: 10.1094/PHYTO-03-10-0090 20822430

[40] Wang G, Liu Z, Lin R, Li E, Mao Z, Ling J, et al. Biosynthesis of Antibiotic Leucinostatins in Bio-control Fungus *Purpureocillium lilacinum* and Their Inhibition on *Phytophthora* Revealed by Genome Mining. PLOS Pathogens. 2016;12: e1005685. doi: 10.1371/journal.ppat.1005685 27416025 PMC4946873

[41] Dong L q., Yang J k., Zhang K q. Cloning and phylogenetic analysis of the chitinase gene from the facultative pathogen *Paecilomyces lilacinus*. Journal of Applied Microbiology. 2007;103: 2476–2488. doi: 10.1111/j.1365-2672.2007.03514.x 18045433

[42] Yang J, Zhao X, Liang L, Xia Z, Lei L, Niu X, et al. Overexpression of a cuticle-degrading protease Ver112 increases the nematicidal activity of *Paecilomyces lilacinus*. Appl Microbiol Biotechnol. 2011;89: 1895–1903. doi: 10.1007/s00253-010-3012-6 21110018

[43] Bonants PJM, Fitters PFL, Thijs H, Belder E den, Waalwijk C, Henfling JWDMY 1995. A basic serine protease from *Paecilomyces lilacinus* with biological activity against *Meloidogyne hapla* eggs. Microbiology. 141: 775–784. doi: 10.1099/13500872-141-4-775 7773385

[44] Xu W-F, Yang J-L, Meng X-K, Gu Z-G, Zhang Q-L, Lin L-B. Understanding the Transcriptional Changes During Infection of *Meloidogyne incognita* Eggs by the Egg-Parasitic Fungus *Purpureocillium lilacinum*. Frontiers in Microbiology. 2021;12. Available: https://www.frontiersin.org/articles/10.3389/fmicb.2021.617710 10.3389/fmicb.2021.617710 PMC8058359 33897634

[45] Xie J, Li S, Mo C, Xiao X, Peng D, Wang G, et al. Genome and Transcriptome Sequences Reveal the Specific Parasitism of the Nematophagous *Purpureocillium lilacinum* 36–1. Front Microbiol. 2016;7. doi: 10.3389/fmicb.2016.01084 27486440 PMC4949223

[46] Park J-O, Hargreaves J r., McConville E j., Stirling G r., Ghisalberti E l., Sivasithamparam K. Production of leucinostatins and nematicidal activity of Australian isolates of *Paecilomyces lilacinus* (Thom) Samson. Letters in Applied Microbiology. 2004;38: 271–276. doi: 10.1111/j.1472-765X.2004.01488.x 15214724

[47] Madariaga-Mazón A, González M, del M, González C, Cerda C, Mata R. Absolute Configuration of Acremoxanthone C, a Potent Calmodulin Inhibitor from *Purpureocillium lilacinum*. Planta Med. 2013;79: s-0033-1348645. doi: 10.1055/s-0033-1348645 23876004

[48] Teles APC, Takahashi JA. Paecilomide, a new acetylcholinesterase inhibitor from *Paecilomyces lilacinus*. Microbiological Research. 2013;168: 204–210. doi: 10.1016/j.micres.2012.11.007 23219197

[49] de Sequeira DCM, Menezes RC, Oliveira MME, Antas PRZ, De Luca PM, Oliveira-Ferreira J de, et al. Experimental Hyalohyphomycosis by *Purpureocillium lilacinum*: Outcome of the Infection in C57BL/6 Murine Models. Frontiers in Microbiology. 2017;8. Available: https://www.frontiersin.org/articles/10.3389/fmicb.2017.01617 10.3389/fmicb.2017.01617 PMC5572354 28878763

[50] Frederiks C, Wesseler JH. A comparison of the EU and US regulatory frameworks for the active substance registration of microbial biological control agents. Pest Management Science. 2019;75: 87–103. doi: 10.1002/ps.5133 29962019 PMC8246847

